# The Week's Parliament and Publications

**Published:** 1910-07-23

**Authors:** 


### The Week's Parliament and Publications.

### THE NOTIFICATION OF PULMONARY PHTHISIS.

Mr. Ure has informed the member for Peebles and Selkirk that up to the present 74 local authorities in Scotland, representing nearly 50 per cent. of the total population, have extended the provisions of the Infectious Diseases (Notification) Act, 1889, to pulmonary phthisis. The Local Government Board proposes shortly to issue a further circular on the subject.

### PHYSICAL TRAINING.

Viscount Hill has introduced a Bill in the House of Lords to provide for physical training in elementary, secondary, and continuation schools. The motion for second reading was withdrawn, but the Bill will probably be reintroduced next year with alterations. The object, as stated in the memorandum to the Bill, is to ensure for each young person the possession of "sound and healthy vital organs, a deep mobile chest, and the development of an evenly balanced muscular and nervous system, and thus to arrest the deplorable physical degeneration disclosed by the report of the Inter-Departmental Committee on Physical Deterioration." It provides that the local education authority shall determine the time to be devoted to training, which must not be less than two hours each week. It also provides for examinations and certificates of proficiency.

### AN UNAUTHORISED EXPERIMENT.

The Return showing the number of experiments on living animals during the year 1909, which was referred to in The Hospital of July 2, mentions the case of a licensee holding certificate B for certain experiments on rabbits who performed an operation which was not authorised by the certificate. The Home Secretary has been asked by Mr. Greenwood for the name of the licensee, but Mr. Churchill has refused to give it, and has explained the circumstances under which the irregular experiment took place. It appears that the licensee, who is a distinguished man of science, held certificates in previous years authorising this very experiment, but he failed to notice that his certificates at the time when he performed the operation were so worded as not to include it.

### UNQUALIFIED MEDICAL AND DENTAL PRACTICE.

Dr. Addison has given notice to ask the Home Secretary if he can state what steps the Government proposes to take, by legislation or otherwise, to protect the public against uneducated and unqualified persons practising medicine or dentistry. Dr. Addison draws special attention to the judicial decision of the House of Lords in the case of Bellerby *v.* Heyworth, and to the Report upon the Administration of Anæsthetics, issued by the Coroners' Committee. The question in the House of Lords case was as to the meaning of the words "specially qualified to practise dentistry" in Section 3 of the Dentists Act, 1878, and in the course of his judgment the Lord Chancellor said the Act itself did not forbid anyone from practising dentistry, but only forbade the assumption of certain titles by unqualified persons implying that they are specially qualified to be registered. In the course of their Report on the Administration of Anæsthetics (Cd. 5111), the Coroners' Committee stated that, as the law stood at present, the administration of anæsthetics was under no regulation. "Although a man cannot sell a glass of beer to another without a licence, he may drug that other person to his heart's content without let or hindrance from the law. Apart from any criminal intent, a bone-setter or a beauty-doctor, or a quack of any kind, is as much at liberty to administer an anæsthetic to his patient for the purpose of an operation as a qualified medical anæsthetist." It will be interesting to hear the Home Secretary's reply, but presumably no action will be taken until the information on the subject of unqualified medical practice, which has been collected by the Local Government Board, has been published.

The Home Secretary has been asked a similar question by Mr. Marshall Hall, and in reply Mr. Churchill has stated that he hopes, when time permits, to propose legislation with regard to the recommendations of the Coroners' Committee.

### WHOOPING-COUGH AND THE M. A. B.

In view of the large aggregate mortality from measles and whooping-cough in London, Sir William Collins has asked the President of the Local Government Board whether he would be prepared to approve of the utilisation of such hospitals of the Metropolitan Asylums Board as may not be required for notifiable diseases, for the voluntary admission and treatment of children suffering from these complaints. Mr. Burns has replied that the question is under consideration, and that a proposal on the subject is coming before the managers of the Metropolitan Asylums District at their next meeting.

### MIDWIVES BILL.

The Midwives (No. 2) Bill, which has been introduced in the House of Lords by Earl Beauchamp, is for the most part founded upon the Report of the Departmental Committee appointed to consider the working of the Midwives Act, 1902 (1909, Cd. 4822), but departs from the recommendations of that Committee in one or two details. It proposes to alter the constitution of the Central Midwives Board, in the direction of increasing the number of the Board from nine to thirteen members, representation being given to the following bodies : The Local Government Board, the Association of Municipal Corporations, the Society of Medical Officers of Health, and the British Medical Association. The member appointed by the last-mentioned association must be a medical practitioner, but no special qualification is prescribed in the case of the members appointed by the other new bodies. Another proposal is that in future the members of the Board appointed by the Incorporated Midwives' Institute and the Royal British Nurses' Association shall be certified midwives. The Bill contains a number of other important proposals, and makes provision for the payment of fees of medical practitioners called in on the advice of midwives. Clause 17 provides that "Where a duly qualified medical practitioner has been summoned upon the advice of a certified midwife attending a woman in child-birth to render assistance in a case of emergency in pursuance of any rule framed by the Central Midwives Board, he shall, on complying with the prescribed conditions, be entitled to recover from the Board of Guardians of the Poor Law Union in which the woman resided such fee in respect of his attendance as may be prescribed." Provision is made for the payment by midwives of an annual registration fee of 1s., and the disciplinary powers of the Central Board are enlarged. The Bill provides for reciprocal treatment of midwives certified in other parts of His Majesty's dominions, and gives local supervising authorities power to contribute to the training of midwives.

